# Automated PD-L1 status prediction in lung cancer with multi-modal PET/CT fusion

**DOI:** 10.1038/s41598-024-66487-y

**Published:** 2024-07-19

**Authors:** Ronrick Da-ano, Gustavo Andrade-Miranda, Olena Tankyevych, Dimitris Visvikis, Pierre-Henri Conze, Catherine Cheze Le Rest

**Affiliations:** 1 LaTIM, UMR 1101, Inserm, University of Brest, Brest, France; 2 Nuclear Medicine, University of Poitiers, Poitiers, France; 3 IMT Atlantique, Brest, France

**Keywords:** Deep learning, Immunotherapy response, Multi-modal fusion, Medical imaging, Applied mathematics, Computational science, Scientific data, Statistics, Predictive markers, Cancer

## Abstract

Programmed death-ligand 1 (PD-L1) expressions play a crucial role in guiding therapeutic interventions such as the use of tyrosine kinase inhibitors (TKIs) and immune checkpoint inhibitors (ICIs) in lung cancer. Conventional determination of PD-L1 status includes careful surgical or biopsied tumor specimens. These specimens are gathered through invasive procedures, representing a risk of difficulties and potential challenges in getting reliable and representative tissue samples. Using a single center cohort of 189 patients, our objective was to evaluate various fusion methods that used non-invasive computed tomography (CT) and $^{18}$F-FDG positron emission tomography (PET) images as inputs to various deep learning models to automatically predict PD-L1 in non-small cell lung cancer (NSCLC). We compared three different architectures (ResNet, DenseNet, and EfficientNet) and considered different input data (CT only, PET only, PET/CT early fusion, PET/CT late fusion without as well as with partially and fully shared weights to determine the best model performance. Models were assessed utilizing areas under the receiver operating characteristic curves (AUCs) considering their 95% confidence intervals (CI). The fusion of PET and CT images as input yielded better performance for PD-L1 classification. The different data fusion schemes systematically outperformed their individual counterparts when used as input of the various deep models. Furthermore, early fusion consistently outperformed late fusion, probably as a result of its capacity to capture more complicated patterns by merging PET and CT derived content at a lower level. When we looked more closely at the effects of weight sharing in late fusion architectures, we discovered that while it might boost model stability, it did not always result in better results. This suggests that although weight sharing could be beneficial when modality parameters are similar, the anatomical and metabolic information provided by CT and PET scans are too dissimilar to consistently lead to improved PD-L1 status predictions.

## Introduction

Lung cancer remains a significant source of disease-related mortality, regardless of the significant therapeutic progress made in recent years. Among the ongoing therapeutic approaches, immunotherapy plays a continuously increasing role in the management of localized or locally advanced tumors, as it stimulates the immune system. The immune system, particularly lymphocytes, are hindered by the immune checkpoint molecules that are expressed by cancer cells. Immunotherapy promotes an effective immune response against the tumor by inhibiting these immune checkpoints. The assessment of immune checkpoint inhibitors, such as programmed cell death-ligand 1 (PD-L1), through techniques like immunohistochemistry (IHC), is considered as a fundamental parameter for the initiation of immunotherapy.

Getting PD-L1 expression status requires surgical or biopsy tumor specimens utilizing obtrusive methods, with an associated chance of morbidities^1,2^. In addition, IHC is tedious and requires high skills from pathologists. Tumor heterogeneity might also influence the precision and reproducibility of PD-L1 assessment using IHC. Another challenge is the potential changes in PD-L1 articulation during treatment, as uncovered in recent studies^3–5^. A non-invasive method for determining PD-L1 status could have a significant impact on clinical decision support given all of these issues, particularly in situations where tissue cannot be accessed or when IHC proves inadequate^6,7^. The established connection between imaging and lymphocytic penetration suggests that the integration of numerous imaging modalities, like positron emission tomography (PET) and computed tomography (CT), may be an alternative for the evaluation of PD-L1 expression status in clinical practice. This is supported by recent studies showing promising results in using radiomics and AI methodology for the extraction of PD-L1 status information from PET/CT images^8–10^.

Currently, PD-L1 expression on tumor cells assessed by immunohistochemistry is the only approved diagnosis biomarker for immunotherapy in patients with NSCLC and has been shown to be related with the efficacy of immune checkpoint inhibitors (ICIs) in NSCLC. In the initial setting, pembrolizumab monotherapy has shown improvements in progression-free survival (PFS) and overall survival (OS) benefits compared to chemotherapy in NSCLC patients with PD-L1 expression on $\ge 50\%$ of tumor cells^11,12^. According to a recent study, though PFS and OS were significantly longer in the pembrolizumab-treated group compared with the chemotherapy-treated group in the full NSCLC cohort with PD-L1$\ge 1\%$, a clear PFS and OS benefit was only observed in the subgroup of patients with PD-L1$\ge 50\%$, with higher PD-L1 expression corresponding to greater benefit^7^. The FDA has since approved IHC examination for PD-L1 status assessment, using a cut-off of 50% tumor proportion score (TPS) for starting treatment with pembrolizumab. Pembrolizumab monotherapy is preferred for patients with stage IV NSCLC and PD-L1 levels of 50% or more who are negative for EGFR mutations and ALK fusions. However, expression levels of PD-L1 are intra-tumorally heterogeneous and dependent on IHC analysis with different antibodies, platforms, as well as multiple scoring criteria, complicating interpretation of the results^13–15^. Within this context, a non-invasive and whole-tumor-based biomarker is urgently needed.

Traditionally, tumors have been characterized in medical imaging using basic standards like size, volume, activity concentration or intensity observed on PET/CT scans respectively. However, with the advent of radiomics strategies^16^, more complex patterns can be characterised by image intensity histograms, texture matrices, or morphological descriptors. The likely advantages of radiomics have been broadly studied in lung cancer^17–20^ where machine learning for patient classification demonstrated its capability in dealing with the large number of parameters from a pre-determined number of patients. Nevertheless, the analysis of radiomics features faces numerous challenges due to the tedious nature of the extraction and computation process. In addition, variability in imaging protocols, scanners, and patient positioning can impact the reproducibility of radiomics features^21,22^. Hence, it is crucial to develop more automated and robust methods for extracting and analyzing radiomic features so that they can be utilized in clinical settings.

Recently, the advancement of learning techniques have been applied in lung cancer screening, assessing drug effectiveness, and enhancing prognosis predictions^23–25^. Specifically, convolutional neural networks (CNNs) are progressively being incorporated into radiomics frameworks. This integration can be accomplished by automating the lesion delineation task using deep convolutional encoder decoder architectures^26–29^, as well as by extracting deep features from intermediate hidden layers. Previous studies have investigated the use of DL for the segmentation and prediction of survival using deep features in NSCLC with $^{18}$FDG PET/CT images^30^. A recent review has summarized the use of DL within the context of detection/classification as well as prediction and prognosis in lung cancer using CT images^31^. Compared to handcrafted features, deep features may contain more representative information and offer more predictive patterns to effectively address the objective tasks. Moreover, deep learning-based approaches can handle complicated and heterogeneous medical imaging data with high effectiveness, making them promising tools for clinical decision-making. Within the context of NSCLC numerous studies have shown the potential of deep features for patient survival assessment or detection of recurrence^28,29,32–34^. More recent studies have shown the potential of deep learning models within the context of radiogenomics. Different studies have shown the potential of predicting PD-L1 expression using CT images, with a relatively good performance (AUC > 0.71)^35–37^. However, all these studies primarily categorized binary patterns on the premise of generally small patient samples (up to 34) based on the usage of CT images only. Combined $^{18}$FDG PET/CT images have been also used in combination with DL to predict EGFR^38–40^ and PD-L1^41^ status for lung cancer, showing improved performance (up to an AUC of 0.85 at the validation stages of the model). However, all these studies have not investigated a comparative performance of different DL architectures or the evaluation of the impact of using multimodality versus single modality imaging within the context of predicting patient genetic profiling. In addition, no studies have explored the impact of different fusion frameworks for the combination of PET and CT images within such DL models.

Our current study aims at exploring the complementary value of PET and CT images within a deep radiomics framework for the prediction of PD-L1 expression in NSCLC. Within the remits of this main objective, various fusion frameworks for combining PET and CT scans within different network architectures were explored. This study was implemented using a single center cohort comprising PET/CT images from 189 NSCLC patients.

## Materials and methods

### Patient cohorts and data collection

The dataset used in this study consists of 189 NSCLC patients having undergone an initial staging PET/CT scan. All images were acquired on a Biograph mCT40 (SIEMENS, Erlangen) using a standard half-body PET/CT imaging protocol based on the EANM guidelines^42^ after intra-venous administration of 2.5–3 MBq/kg of $^{18}$F-FDG. A low-dose CT was acquired for attenuation correction and anatomical correlation of PET abnormalities (tube current 120 kV, CARE Dose 4D current modulation system, reconstruction using 5 mm slice thickness). PET acquisitions were performed using 3.5 minutes per bed position, and PET images were reconstructed using the OSEM-TrueX-TOF algorithm (3 iterations, 21 subsets, voxel size of 4.073$\times$4.073$\times$2.072 mm$^3$). In addition to images, the dataset includes patient clinical attributes including factors like sex, stage, age, and PD-L1 expression levels. Out of the total 189 cases, 105 cases (56%) were assigned as PD-L1 positive, while the remaining 84 cases (44%) were categorized as PD-L1 negative.

###  Data pre-processing and model training

To guarantee consistency among PET and CT volumes, PET images were resized to match the CT scans for every patient. This resizing procedure includes applying bilinear interpolation methods, ensuring the preservation of the original content of the image while accomplishing dimensional alignment. We preferred to avoid downsampling of the CT images to the PET resolution based on the EANM/SNMMI recommendations^43^ where it is clearly mentioned that downsampling may lead to aliasing artifacts and may require application of a low-pass filter prior to resampling. At the same time they have noted that there are no clear indications whether upsampling or downsampling schemes are preferable. The alternative would have been to use both upsampling and downsampling of the PET and CT images to a common isotropic voxel size of, for example, $2 \times 2 \times 2$ mm$^{3}$ but this configuration was not tested in this work. In order to facilitate targeted whole lung volume analysis, we developed a whole lung segmentation model. To accomplish this, we used the U-Net model^44^ trained by utilizing the publicly accessible LIDC-IDRI dataset which includes 1018 cases^45^. Each case of this dataset includes clinical thoracic CT images and an XML file containing the outcomes of a two-phase image annotation process conducted by four experienced thoracic radiologists. The trained U-Net model was subsequently used to segment the whole lung fields in the CT images of our patient datasets.

**Figure 1:**
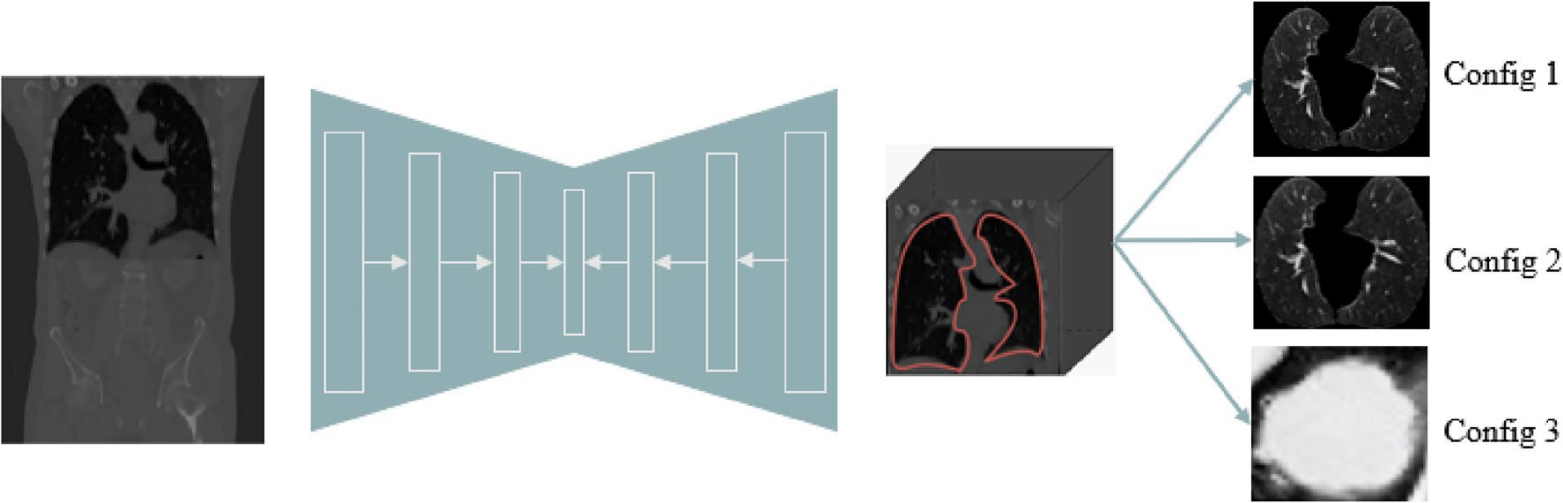
ROI: Lung segmentation using UNet Model trained on LIDC-IDRI to obtain 3 different PET/CT image configurations.

The segmented lung fields were used to define different image configurations as input to the network model architectures and fusion schemes of the PET and CT images (Fig. 1). The initial setup, denoted as Config1, considers a bounding box of 10 pixels in all three dimensions around the segmented whole lung field. This configuration was considered to account and evaluate the potential impact of any expected errors in segmentation while guaranteeing total coverage of the lung region. The second configuration, denoted as Config2, utilizes a larger, broadly extended bounding box with an extra margin of 100 pixels, which incorporates lung regions as well as surrounding tissues. This configuration allows assessing the potential impact of avoiding the whole lung segmentation step using instead as input to the network most of the original acquired images. The last configuration denoted as Config3, concerns the largest lung lesion semi-automatically segmented using the Fuzzy Locally Adaptive Bayesian (FLAB) algorithm^46,47^. The obtained volume of interest was adjusted manually by an experienced clinician. The performance of this algorithm has been extensively evaluated for functional tumor volume segmentation, demonstrating high reproducibility and robustness for different cancer models^35–37,47^. Tumor segmentation on CT images was performed by applying the PET tumor volume of interest on CT images using 3D Slicer^48^. These configurations allow us to comprehensively assess the impact of the tissue/organ types, including tumor only, used as an input to the models for the PD-L1 prediction.

We executed our models utilizing PyTorch and MONAI (https://monai.io/). Various models with relating configurations were prepared using Nvidia A6000, Titan RTX and 2080 GPUs. For our models, we utilized a standardized image size of 90$\times$90$\times$90 on each of the six data pipelines and performed noise reduction for each modality, as displayed in Table 1. To reduce overfitting, data augmentations such as horizontal flip, random resizing cropping, random rotation, and random color jittering were used throughout the training phase. The model was hence trained and evaluated five times, each time involving a different fold as the validation set. This approach allows us to assess the performance and over-simplification of the model on various data subsets exhaustively.

All models were trained for a sum of 200 epochs, with a batch size of 1. Different optimizers and learning rates were chosen relying upon the model architecture: the DenseNet-264 and EfficientNetBN1 backbones used the Stochastic Gradient Descent (SGD) with a learning rate (LR) of 0.001. The decision to use SGD for these designs originates from its adequacy in taking care of complex models with numerous parameters, like DenseNet and EfficientNet. A learning rate of 0.001 was considered satisfactory to guarantee smooth albeit with a slower convergence. For the ResNet-101 backbone, we used the Adam optimizer with a learning pace of 0.01. Somewhat higher learning rate contrasted with different backbones favors quicker convergence due to the generally less cumbersome ResNet model, in contrast to DenseNet or EfficientNet.

To additionally work on the model’s convergence, we utilize a step decay methodology in changing the learning rate. We applied step decay with a component of 20 for the ResNet model, while an element of 10 was used for DenseNet and EfficientNet. This approach gradually reduces the learning rate during training, working with critical updates during the initial phases of training and better. The training target used was a cross-entropy loss function while utilizing a few on-the-fly data augmentation methods including rotation, flipping, zooming, and random shift intensity.

**Table 1:** Hyper-parameters used for the different pipelines.

BackBone	Initial LR	Dynamic LR	Dimensions	Optimizer	Loss	Batch Size	Architectures
ResNet	0.01	Yes	90$\times$90$\times$90	Adam	CrossEntropy	1	All
DenseNet	0.001	Yes	90$\times$90$\times$90	SGD	CrossEntropy	1	All
EfficientNet	0.001	Yes	90$\times$90$\times$90	SGD	CrossEntropy	1	All

### Network architectures

In this work, we investigate the use of diverse deep learning models and multi-modal fusion techniques for automated prediction of PD-L1 status in lung cancer utilizing both PET and CT imaging modalities. To achieve this goal, we consider three broadly used CNN backbones: ResNet101, DenseNet264 and EfficientNetB1. These models were chosen because they are well-known and widely used in the field of deep learning for various computer vision tasks, including image classification, object detection, and segmentation. They have demonstrated state-of-the-art performance on benchmark datasets and are known for their effectiveness in learning complex patterns and features from images. ResNet101 and DenseNet264 are deep convolutional neural network architectures with a large number of layers. They are capable of capturing complex features and representations from images, which may be beneficial for tasks requiring detailed analysis of medical images such as PET and CT scans. EfficientNetB1, on the other hand, is known for its efficiency in terms of computational resources while maintaining competitive performance.

ResNet101: Residual Network (ResNet) forms a CNN architecture built on the concept of residual learning^49^. This strategy encourages the training of deep neural networks by presenting short-cut connections that skip one or more layers.

DenseNet264: Densely connected convolutional network (DenseNet) is another CNN model known for its densely connected layers^50^. Each layer is firmly connected to all others in a feed-forward way, ensuring a solid level of feature reuse and a consistent stream of information across the network.

EfficientNetB1: A more recent expansion to the CNN architectures, EfficientNet is outlined for principled scaling, including not only the expansion of layer depth and width but also the determination of the input^24^.

In spite their distinct characteristics, these models take a comparative operating system. In each model, information passes through a sequence of convolutional layers, where each layer incrementally extracts higher-level feature representations. After passing through convolutional layers, a subsequent global average pooling layer is presented. This pooling layer decreases spatial dimensionality while preserving basic information. Subsequently, a fully connected layer maps the high-dimensional information into likelihood scores for each class. The sigmoid function is then applied to these scores to yield a final classification. This sequential stream of information from the initial input to the final classification, is a shared characteristic common to all three models, empowering consistent interchangeability while keeping the overall architecture. Leveraging these models, we design six distinctive data architectures that consider both single and multi-modal fusion procedures.

We characterize the performance of different models and associated fusion protocols using PET and CT modalities. Here, we denote $\pmb {I}_{\texttt {CT}} \in {\mathbb {R}}^{H\times W \times D}$ and $\pmb {I}_{\texttt {PET}} \in {\mathbb {R}}^{H\times W \times D}$ the PET and CT images respectively. Our principal objective is to develop a deep learning model predicting PD-L1 classification, denoted as $y \in \{0, 1\}$, in view of fused PET/CT images. For this purpose, we employ CNN-based models, which process the modalities either independently or jointly, depending upon the chosen fusion scheme. In both scenarios, we utilize models with associated weights $\Theta$, to extract features from the input images, resulting in a high-level feature representation $\pmb {h}_{i} = f(\pmb {I}_{i}; \Theta )$ with $i \in \{\texttt {CT},\texttt {PET}\}$.

In cases adopting a late fusion scheme, we derive two feature vectors, $\pmb {h}_{CT}$ and $\pmb {h}_{PET}$, obtained by processing $\pmb {I}_{\texttt {CT}}$ and $\pmb {I}_{\texttt {PET}}$ independently through their respective model networks. These feature vectors are then linked and concatenated through a fully associated connected (FC) to deliver the last prediction ${\hat{y}}=g(\pmb {h}_{CT}, \pmb {h}_{PET})$, where *g* refers to the FC layer, outfitted with a different arrangement of weights.

We aim to determine the set of optimal weights $\Theta$ and the weights of the FC layer which minimize the cross-entropy loss between the predicted class ${\hat{y}}$ and the ground truth class variant *y* on an informational index of size *N*. This is mathematically formalized as the minimization of the following loss function:

1 $$\begin{aligned} \displaystyle \min _{\Theta } \sum _{n=1}^{N} {\mathscr {L}}(y_n, {\hat{y}}_n) \end{aligned}$$

where ${\mathscr {L}}(y, {\hat{y}}) = - y \log ({\hat{y}}) - (1-y) \log (1-{\hat{y}})$ is the binary cross-entropy loss. This problem formulation outlines the way we handle the multi-modal data, highlighting the fusion of PET and CT image modalities for improved PD-L1 status classification.

#### Architecture A

**Figure 2:**
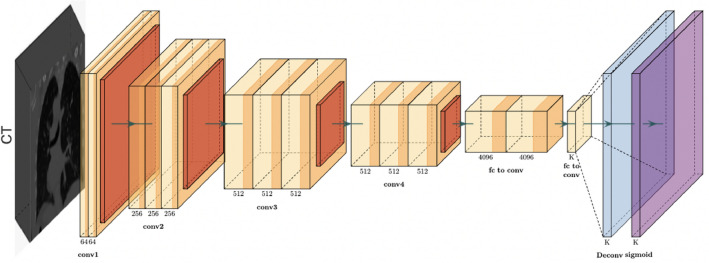
Architecture A: using only CT images as an input to the model.

This architecture exclusively depends on CT scans as input information (Fig. 2). This backbone comprises of few layers that steadily abstract the input information into high-level features which follows the data pre-processing and model training section. A global average pooling layer follows to decrease the spatial dimensions while keeping the most important information. These high-level features are then forwarded to completely connected layers that execute the mapping into scores for each class. A final classification is created by applying these scores to the softmax function. This cycle addresses the complete stream of information within this architecture, from the input CT volume to the final PD-L1 status forecast.

#### Architecture B

**Figure 3:**
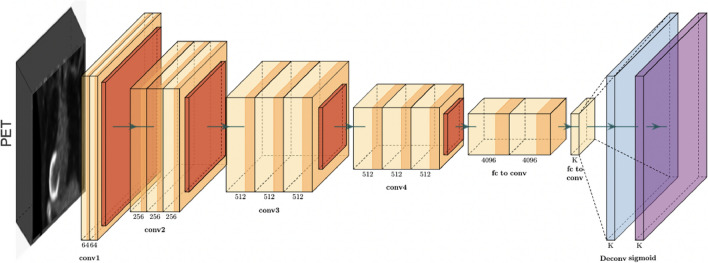
Architecture B: using only PET images as an input to the model.

Comparable to the previous architecture, Architecture B serves as a baseline model focusing only on the PET (Fig. 3). This architecture is trained exclusively on PET images, utilizing an isolated assessment of the individual contributions of PET volumes to PD-L1 status prediction. The designated CNN-based models that are similar to Architecture A are fed into the data following the same data preprocessing. By directing a comparative investigation of the performances of Architecture A and B, we can effectively evaluate and separate the predictive capacities of PET and CT modalities.This comparative evaluation provides insights into the specific and individual contributions of each modality to the task of PD-L1 status prediction. After performing the same information pre-processing, it is fed into the assigned CNN-based models comparative to Architecture A. By conducting a comparative investigation of the exhibitions of Architecture A and B, we will viably evaluate and separate the prescient capabilities of PET and CT modalities.

#### Architecture C

**Figure 4:**
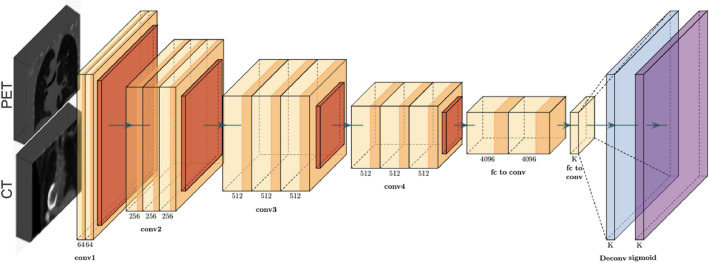
Architecture C: using both PET and CT images as an input to the model within an early fusion framework.

The C architecture considers an early fusion approach, combining the PET and CT modalities right at the beginning of the processing pipeline (Fig. 4). The fundamental stage incorporates partitioned data pre-processing steps for PET and CT information. These preprocessed PET and CT volumes are at that linked together, making a consolidated input to a single CNN-model. The C architecture is outlined to investigate the advantages of early fusion, aiming to choose if a jointly learned representation outperforms models that depend on reasonable a single modality. By conducting a comparative examination between architecture C and its counterparts, A and B, assessing architecture C intends to explain the impact of early fusion on the predictive capabilities of the classification pipeline.

#### Architecture D

**Figure 5:**
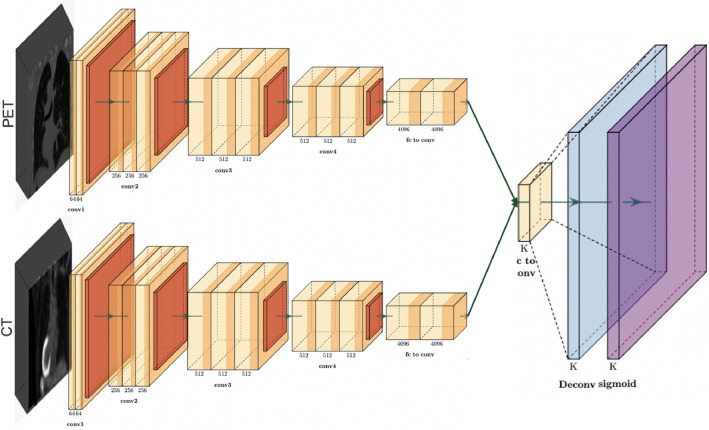
Architecture D: using both PET and CT images as an input to the model within a late fusion framework and without shared weights representation.

Architecture D proposes a late fusion technique, as shown in Fig. 5. After performing data pre-processing for PET and CT data, the volumetric information from the two modalities are coordinated independently to their partitioned CNN models. These models are trained to extract and capture pertinent data from the input information, eventually creating high-level features. In this pipeline, the models learn modality-specific representations, without inter-modality conditions. The fusion of modality-specific deep feature vectors from both PET and CT modalities occurs prior to their presentation to fully connected layers. This late fusion framework allows the network to explore the specific information from each modality independently. The final step of this architecture is to apply a sigmoid to the likelihood scores created from the connected layers to provide the PD-L1 status.

#### Architecture E

**Figure 6:**
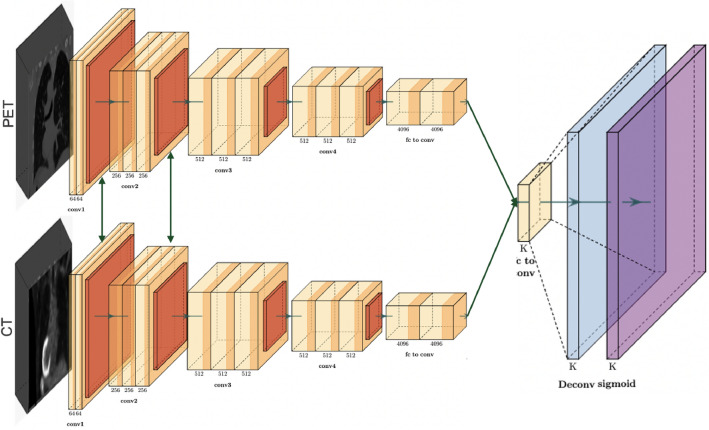
Architecture E: using both PET and CT images as an input to the model within a late fusion framework with partially shared weights representation.

Architecture E closely resembles Architecture D, with the principal distinction being that specific layers in the configuration share similar weights, as displayed in Fig. 6. This strategic approach leverages the existence of shared low-level features that are present across various modalities. The shared component incorporates the initial segment of the network, responsible for processing features such as edges and textures. As the network goes further into the last layers, we will keep up with their separate weight sets, permitting them to completely learn and separate higher-level, task-specific features. These high-level features frequently incorporate more complex and unique information perspectives that are straightforwardly connected with the particular tasks at hand. When the feature extraction stage is finished, fully connected layers are applied, followed by a sigmoid function that characterizes the PD-L1 status. This shared and separate weighting framework can be applied to various model structures, with varieties in the particular common classes implement relying upon the design and requirements of individual models.

#### Architecture F

**Figure 7:**
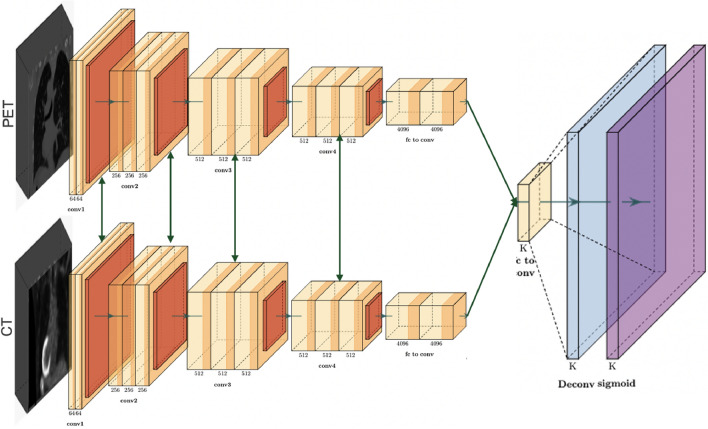
Architecture F: using both PET and CT images as an input to the model within a late fusion framework with fully shared weights representation.

The architecture F, outlined in Fig. 7, addresses the last part of weight sharing among similar networks. Compared to the E architecture, which utilizes constrained weight sharing, this approach receives a strongly all-weight-sharing procedure. The outcome of this architecture could be a synchronized feature extraction process for both modalities. In this course of action, the PET and CT volumes experience isolated data pre-processing steps before entering the models, which is completely shared-weight. These models are responsible for feature extraction, pooling, and following fully connected layers. Finally, the sigmoid function prompts the classification of PD-L1 status. Architecture F aims to make a universal and versatile representation able of capture shared traits over the PET and CT modalities. By differentiating this architecture to the already depicted models in our study, we aim to understand the potential performance benefits displayed by complete weight sharing to diverse structures.

### Performance evaluation

During the training and validation stage, we utilized a random choice of samples to ensure the diversity in characteristics of the dataset. In each fold, we utilize 132 samples for training, and 57 for testing, yielding a split of 70% and 30%, separately. The uneven partitioning of samples between the training and testing sets is reused across all folds, and the final model performance estimate was determined by averaging the results obtained from each fold.

The Area Under the Curve (AUC) metric and Receiver Operating Characteristic (ROC) curve analysis, sensitivity and specificity were used to assess the performance of the classifiers. The DeLong strategy was utilized to determine the 95% confidence intervals (CI). The median and interquartile range (IQR) with 95% CI were utilized to address continuous variables. All statistical tests were two-tailed, with statistical significance set at p < 0.05.

###  Ethical standards and informed consent

 The study was approved by the local ethics committee of the University Hospital Poitiers, France. Informed consents were obtained for all patients. All procedures were in accordance with the ethical standards of the institutional research committee and with the 1964 Helsinki declaration and its later amendments.

## Results

The mean AUC, specificity, and sensitivity of various models across various architectures, as well as their 95% confidence intervals in testing datasets, are the primary focus of this section, which provides a comprehensive overview of their performance.

In Config 1 as displayed in Table 2, for ResNet, the architecture C showed a better performance with an AUC of 0.79 with a confidence interval of (0.77–0.81), which is generally narrow, showing a high level confidence in this result. Architecture B of ResNet additionally shows better performance with an AUC of 0.75 (CI: 0.72–0.77), comparable to architecture E. These architectures are reliably associated with high AUC values. In addition to that, ResNet and DenseNet models maintain robust AUC scores across various architectures (C to F), recommending that it is a reliable decision for this classification task. Specificity and sensitivity values are fairly consistent across architectures, demonstrating that DenseNet offers balanced performance across different classes. EfficientNet architecture D performs well, with AUC scores of 0.77 (CI: 0.74–0.79). While not as high as ResNet or DenseNet, these values indicate competitive performance. Specificity and sensitivity values for EfficientNet are relatively consistent, showing balanced class prediction.

**Table 2:** Results for different involved architectures and data pipelines using the testing datasets in Config1. Significant values are in bold underline.

Model	Architecture	Mean AUC (95% CI)	Mean specificity (95% CI)	Mean sensitivity (95% CI)
ResNet	A	0.73 (0.70–0.75)	0.71 (0.70–0.74)	0.72 (0.70–0.75)
	B	0.75 (0.72–0.77)	0.70 (0.70–0.73)	0.71 (0.70–0.74)
	C	**0.79** (0.77–0.81)	**0.75** (0.72–0.77)	**0.77** (0.74–0.79)
	D	0.70 (0.69–0.74)	0.73 (0.71–0.77)	0.73 (0.71–0.76)
	E	0.75 (0.72–0.78)	0.74 (0.71–0.78)	0.74 (0.72–0.77)
	F	0.74 (0.71–0.76)	0.74 (0.71–0.76)	0.74 (0.71–0.76)
DenseNet	A	0.71 (0.70–0.74)	0.70 (0.70–0.72)	0.71 (0.70–0.74)
	B	0.78 (0.76–0.81)	0.71 (0.71–0.73)	0.72 (0.70–0.73)
	C	**0.80** (0.78–0.82)	**0.76** (0.74–0.78)	**0.76** (0.74–0.79)
	D	0.78 (0.77–0.82)	0.73 (0.71–0.75)	0.74 (0.72–0.76)
	E	0.77 (0.76–0.80)	0.75 (0.73–0.77)	0.73 (0.71–0.75)
	F	0.75 (0.72–0.76)	0.74 (0.72–0.76)	0.74 (0.72–0.77)
EfficientNet	A	0.73 (0.73–0.77)	0.72 (0.70–0.74)	0.71 (0.70–0.75)
	B	0.75 (0.73–0.78)	0.71 (0.70–0.74)	0.71 (0.70–0.73)
	C	0.73 (0.70–0.75)	0.74 (0.72–0.76)	0.73 (0.71–0.75)
	D	**0.77** (0.74–0.79)	0.74 (0.72–0.77)	0.73 (0.70–0.74)
	E	0.76 (0.75–0.79)	0.73 (0.71–0.74)	**0.74** (0.71–0.76)
	F	0.73 (0.70–0.75)	**0.75** (0.72–0.77)	0.73 (0.71–0.74)

Table 3 presents the assessment of the various models using the extended whole lung field image configuration (Config 2). Architecture C provides the better performance with an AUC of 0.80 (95% CI: 0.78–0.83). It provides consistently good results across AUC, specificity, and sensitivity metrics. ResNet architecture D and E display mixed performance, with AUC values of 0.76 and 0.73, respectively. Regardless of contrasts in AUC, the models perform similarly in terms of specificity and sensitivity, with values around 0.72. The DenseNet C architecture demonstrates robust performance with an AUC of 0.81 (95% CI: 0.80–0.85), similarly to the performance of the ResNet C architecture. These results show that the C architecture consistently performs well when it comes to binary classification. DenseNet’s architecture D also performs well with an AUC of 0.79 (95% CI: 0.78–0.83). Like ResNet, DenseNet keeps a harmony among explicitness and responsiveness. DenseNet structures E and F yield serious AUC upsides of 0.76 and 0.74, separately. Even though these values are slightly lower than those of the best architectures, they still provide balanced performance for class prediction. EfficientNet’s architecture D performs better with an AUC of 0.79 (95% CI: 0.78–0.82).

**Table 3:** Results for different involved architectures and data pipelines using the testing datasets in Config2. Significant values are in bold underline.

Model	Architecture	Mean AUC (95% CI)	Mean specificity (95% CI)	Mean sensitivity (95% CI)
ResNet	A	0.72 (0.70–0.74)	0.70 (0.70–0.74)	0.71 (0.70–0.73)
	B	0.76 (0.74–0.79)	0.73 (0.71–0.74)	0.72 (0.70–0.73)
	C	**0.80** (0.78–0.83)	**0.76** (0.73–0.77)	**0.76** (0.74–0.78)
	D	0.76 (0.74–0.79)	0.75 (0.72–0.77)	0.75 (0.73–0.77)
	E	0.73 (0.70–0.74)	0.72 (0.70–0.73)	0.72 (0.70–0.73)
	F	0.72 (0.70–0.73)	0.71 (0.70–0.74)	0.71 (0.70–0.74)
DenseNet	A	0.75 (0.72–0.77)	0.72 (0.70–0.75)	0.71 (0.70–0.74)
	B	0.73 (0.70–0.76)	0.73 (0.71–0.75)	0.72 (0.70–0.75)
	C	**0.81** (0.80–0.85)	**0.78** (0.75–0.80)	**0.76** (0.73–0.79)
	D	0.79 (0.78–0.83)	0.77 (0.75–0.80)	0.75 (0.72–0.77)
	E	0.78 (0.75–0.81)	0.73 (0.70–0.75)	0.74 (0.73–0.78)
	F	0.75 (0.73–0.78)	0.73 (0.71–0.74)	0.75 (0.72–0.78)
EfficientNet	A	0.73 (0.71–0.77)	0.72 (0.70–0.73)	0.72 (0.70–0.75)
	B	0.78 (0.76–0.81)	0.73 (0.70–0.76)	0.71 (0.70–0.73)
	C	0.73 (0.70–0.75)	0.71 (0.70–0.74)	0.73 (0.70–0.76)
	D	**0.79** (0.78–0.82)	**0.78** (0.76–0.80)	**0.77** (0.74– 0.79)
	E	0.76 (0.74–0.79)	0.74 (0.72–0.78)	0.73 (0.71–0.76)
	F	0.74 (0.71–0.76)	0.74 (0.71–0.77)	0.75 (0.72–0.78)

Table 4 presents the assessment of the different models while utilizing the tumor segmented volumes as only input. Architecture C yields the better performance with an AUC of 0.83 (95% CI: 0.81–0.86). The C architecture provides reliably good results across AUC, specificity, and sensitivity metrics. ResNet architecture D and E show mixed performance, with AUC values of 0.77 and 0.74, respectively. Regardless of contrasts in AUC, the models perform similarly in terms of specificity and sensitivity, with values around 0.73. The DenseNet C architecture illustrates robust performance with a best mean AUC of 0.84 (95% CI: 0.82–0.88), similarly to the performance of the ResNet C architecture. These results highlight the consistently good performance of the C architecture for binary classification. Architecture D of DenseNet also performs well with an AUC of 0.80 (95% CI: 0.77–0.81). Comparative to ResNet, DenseNet maintains a balance between specificity and sensitivity. DenseNet architectures E and F have AUC values of 0.80 and 0.76, respectively, that are competitive. The EfficientNet’s architectures D and E architectures perform better with AUCs of 0.83 (95% CI: 0.81–0.83) and 0.77 (95% CI: 0.74–0.80), respectively, despite being slightly lower than the architectures that perform the best in class prediction.

**Table 4:** Results for different involved architectures and data pipelines using the testing datasets in Config3. Significant values are in bold underline.

Model	Architecture	Mean AUC (95% CI)	Mean specificity (95% CI)	Mean sensitivity (95% CI)
ResNet	A	0.73 (0.72–0.75)	0.72 (0.71–0.75)	0.72 (0.70–0.75)
	B	0.78 (0.76–0.81)	0.75 (0.73–0.78)	0.74 (0.72–0.77)
	C	**0.83** (0.81–0.86)	**0.79** (0.76–0.81)	**0.77** (0.75–0.80)
	D	0.77 (0.74–0.79)	0.75 (0.72–0.77)	0.76 (0.74–0.78)
	E	0.74 (0.72–0.76)	0.73 (0.70–0.75)	0.72 (0.70–0.73)
	F	0.73 (0.70–0.75)	0.71 (0.70–0.74)	0.73 (0.71–0.75)
DenseNet	A	0.76 (0.73–0.78)	0.74 (0.72–0.76)	0.73 (0.71–0.76)
	B	0.72 (0.71–0.74)	0.73 (0.71–0.75)	0.72 (0.70–0.75)
	C	**0.84** (0.82–0.88)	**0.80** (0.78–0.83)	**0.78** (0.75–0.81)
	D	0.80 (0.78–0.83)	0.76 (0.75–0.80)	0.76 (0.74–0.79)
	E	0.80 (0.77–0.81)	0.73 (0.70–0.75)	0.73 (0.73–0.78)
	F	0.76 (0.75–0.79)	0.75 (0.72–0.77)	0.76 (0.73–0.78)
EfficientNet	A	0.78 (0.76–0.80)	0.74 (0.72–0.75)	0.73 (0.70–0.75)
	B	0.80 (0.79–0.82)	0.75 (0.73–0.77)	0.73 (0.71–0.74)
	C	0.74 (0.72–0.76)	0.73 (0.72–0.76)	0.73 (0.70–0.76)
	D	**0.83** (0.81–0.86)	**0.80** (0.78–0.82)	**0.80** (0.78– 0.81)
	E	0.77 (0.74–0.80)	0.75 (0.73–0.78)	0.74 (0.72–0.76)
	F	0.74 (0.72–0.76)	0.75 (0.73–0.77)	0.76 (0.74–0.78)

Among all the models assessed, Architecture C outperforms the rest of the configurations, highlighting the significance of the architecture in improving the overall performance of the model. This finding highlights the significance of the Architecture C in improving the overall performance of the model. In terms of AUC, ResNet and DenseNet typically perform better than EfficientNet, suggesting that they might be better suited for this particular classification task. For both Architecture F of ResNet and Architecture C of DenseNet, narrow CIs indicate results that are more reliable and consistent. In looking over the performance of the ResNet, DenseNet and EfficientNet models over different architectures, we observe that early fusion reliably yields better outcomes relative to a late fusion as appeared by the mean AUC results, illustrating better model performance across diverse configurations. Interestingly, among the late fusion variations (D, E, F), it is the architecture D, where loads are not shared, that performs better than the models E and F that utilize loads’ sharing. This observation suggests that not sharing weights in the late fusion cycle may be an ideal setup, recommending a degree of freedom within the basic features captured by the PET and CT images

**Figure 8:**
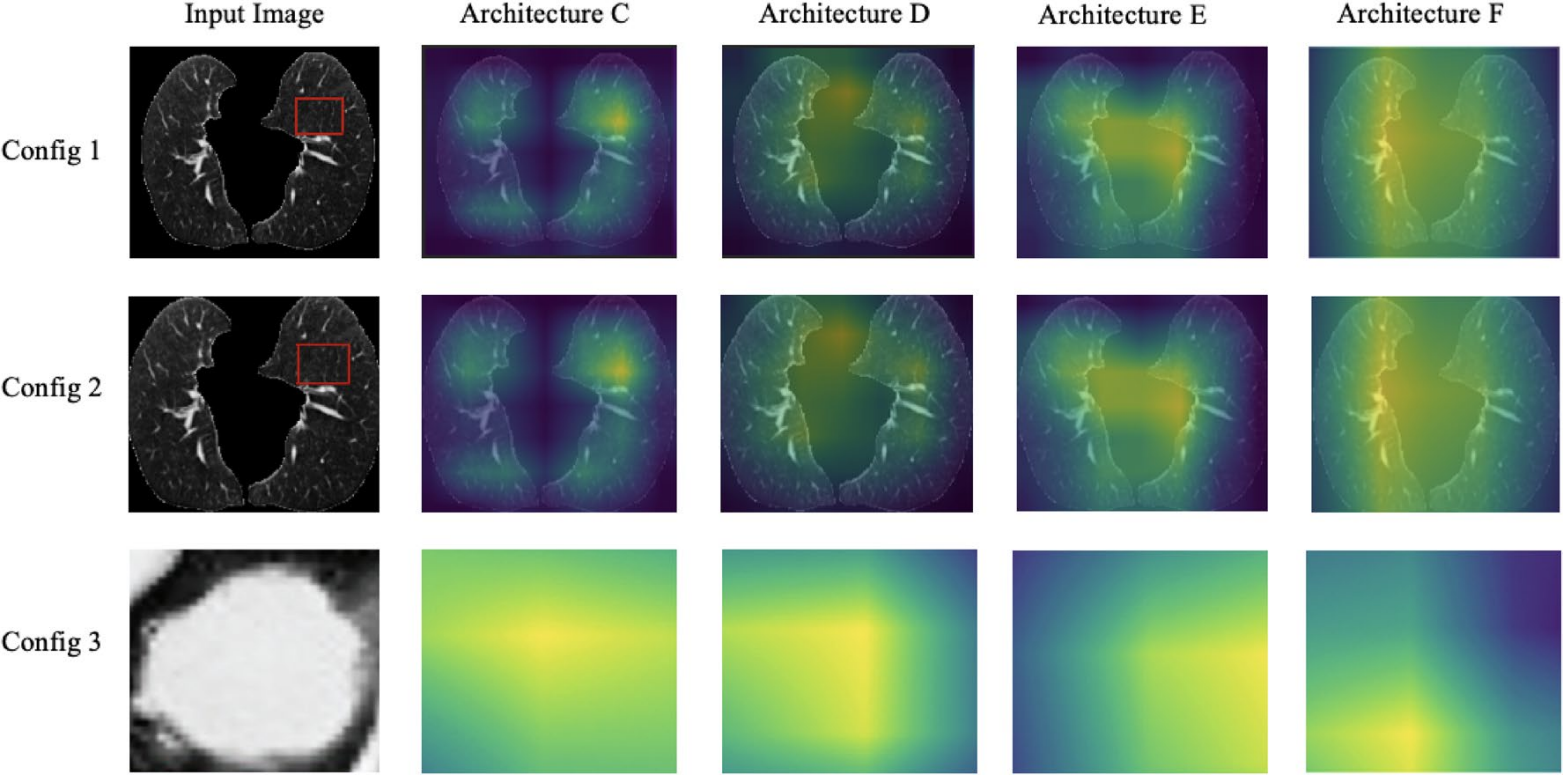
Deep learning feature heatmap representation of architectures C, D, E, and F for predicting PD-L1 expression in Configs 1–3.

All three configurations in terms of the image data volumes used as an input (see Fig. 1), illustrate comparable performance across the ResNet, DenseNet, and EfficientNet models, with certain architectures consistently outperforming others. Config 3 shows better performance compared to Configs 1 and 2, this is likely due to that fact that this configuration is focused on the specific lesion segmentation. Besides, the early fusion architecture demonstrates a more modest standard deviation than those seen in the late fusion architectures.This recommends a more consistent model execution, with less variability between different runs, further supporting the quality and robustness of the early fusion architecture. Taken together, these results highlight the predominance of early fusion over late fusion, the adequacy of not sharing weights during late fusion, and predictable and dependable execution unwavering performance given by the early fusion technique. The classification activation map (CAM) visualization of the predictive model identified the center of the tumor region as a critical region for PD-L1 status classification (Fig. 8).

## Discussion

The principal objective of this study concerns the classification of the PD-L1 status in NSCLC patients utilizing deep learning in combination with multi-modality PET/CT images. A specific secondary objective concerns the evaluation of different configurations spanning from the DL model choice to the input images used including late or early fusion of information provided from functional and anatomical images. This approach varies from the investigations in the current literature, in which deep learning models and input conditions are regularly chosen first and subsequently enhanced with clinical or radiomic data.

Imaging as a noninvasive method is highly effective in predicting gene expression and molecular levels in many types of tumors^51,52^ and may serve as an important surrogate marker in treatment decision-making. The current state of the art in lung cancer immunotherapy involves significant progress, with immunotherapy emerging as a crucial therapeutic approach^53,54^ . In the case of lung cancer and to predict PD-L1 expression, to the best of our knowledge, there are only limited studies focused on predicting PD-L1 expression in their vast majority based on the use of PET/CT images^55,56^. Chest CT examination is the most routine detection method in the process of lung cancer diagnosis and treatment, as it is non-invasive, convenient, and easy to perform in daily clinical routine. Almost all NSCLC patients undergo multiple CT scans to track the progression of tumor lesions. On the other hand, in recent years, AI technology, especially deep learning, has been widely used for the interpretation of medical images. Deep learning technology has endless potential for early detection, diagnosis, and treatment of lung cancer, from detecting lung nodules to identifying benign and malignant nodules in the lungs to further subtyping^57–59^. However, challenges persist, especially in assessing genetic tumor characterisation, including PD-L1 expression, through non-invasive methods^60–63^.

A non-invasive, deep learning-based PET/CT multi-modality imaging-based strategy is proposed in this study to address these difficulties. The examinations include a critical consideration for model execution, given a chosen architecture and comparing input information. More explicitly, a relative investigation of state-of-the-art networks such as ResNet, DenseNet, and EfficientNet in this setting addresses a novel contribution to the field. The integration of multi-modality imaging and the proposed fusion systems likewise showcase advancements beyond what has been investigated in past studies, underlining the novelty and significance of the ongoing work.

The ResNet architecture highlighted its suitability for PD-L1 expression prediction, as evidenced by its consistently superior performance. Besides the equivalent performance of the two fusion strategies in terms of AUC, the higher stability shown by architecture C across various folds demonstrates the superior generalisation capabilities of the early fusion approach. The preference for early fusion over late fusion indicates that integrating information from both PET and CT modalities at an early stage enhances model performance. The expected advantage of early fusion lies in its capacity to give a proper representation of PET and CT data at lower levels of the network, in this way permitting complex designs. The study also explored different fusion scenarios considering the temporal context of combining information recovered from functional and anatomical images, including weight sharing. Our outcomes also show that weight sharing may not be appropriate, considering that different type of information is given by the two imaging modalities considered (metabolism for $^{18}$F-FDG images and anatomy for the CT images). This allows for a degree of freedom when it comes to capturing fundamental features from PET and CT images, while also highlighting the significance of not sharing weights during late fusion. Optimizing fusion strategies for better PD-L1 expression prediction can benefit from these findings. The relationships between these types of data are complementary, and as such the choice to share weights depends on the particular characteristics of the data.

Considering the results for models based on a single image input, the PET-only architecture shows better performance when using DenseNet, while ResNet is best with CT only images. In terms of absolute performance our results (mean AUC from 0.71 to 0.75 depending on the network used) are similar to those achieved by previous studies using CT images only for PD-L1 prediction. All of the single image configurations performed consistently worse than the networks using the multi-modality images, clearly demonstrating the interest of using both PET and CT images for this specific task.

The choice of the input image bounding box size, was also considered with respect to easiness of use and computational efficiency. Different input image configurations, such as whole lung segmented volumes, 2 times extended whole lung configurations, and tumor segmented volumes, were evaluated. The best results were consistently achieved with the use of the tumor only volumes, irrespective of the network, fusion or combination of image inputs considered. This clearly demonstrates that as expected there is no information concerning the PD-L1 status to be derived by using the whole lung volumes including healthy tissue, which generally led to worst performance. This result suggests that if one choses to use the whole images as an input to the network, there is a need to incorporate an automatic tumor segmentation from both the PET and CT images as a first phase of the network before proceeding to the extraction of deep features and their early fusion.

The essential limitation of our study is the lack of multi-center data but also limited dataset size which may influence the performance of the late fusion architecture. Future research directions will involve validation of this single center study results with external datasets within a multi-center study. While our findings offer valuable insights into the effectiveness of various architectures and fusion combinations in PD-L1 status classification, further investigation is warranted, particularly focusing on recent fusion methodologies. More specifically, recent studies^27–29^ have shown that the combination of hand-crafted radiomics features within deep learning models can lead to enhanced results compared to image only models. Future investigations within the objective of further improving the obtained performance of deep learning models for PD-L1 prediction in NSCLC patients, will clearly need to include alternative fusion strategies, such as for example the fusion of deep learning features with hand-crafted features at an early or late stages.

## Conclusion

While concatenating PET and the CT image vectors as proposed in this work is one way to deal with integrating the two modalities into the model, utilizing a double channel encoding procedure could offer benefits regarding effortlessness and interpretability. The two procedures have their benefits and might be more reasonable in various settings. The concatenation approach offers adaptability in how the model learns how to coordinate PET and CT data, considering more complicated cooperations between the modalities. However, it might also add complexity and additional parameters to the model architecture. The optimization of a deep learning model framework for the combination of PET and CT images in the context of PD-L1 expression prediction in NSCLC is what makes our research significant. By integrating information from these two imaging modalities, our study shows a significant enhancement in the accuracy of PD-L1 expression prediction relative to the use of a single modality only. Besides, the investigation of different fusion schemes for PET and CT image integration within diverse network architectures suggests that an early fusion leads to the best performance. In addition, the best results were obtained by focusing on the analysis of the primary lung lesion rather than the whole lung field. Based on these findings our study adds significant knowledge to the understanding of optimal techniques for leveraging multimodal imaging information in predictive modeling of PD-L1 status in NSCLC. PD-L1 status is also important in other cancer types and as such the use of the same models may also play a role in predicting PD-L1 status in other cancers.

## Data Availability

PET/CT data can be made available on request and with permission of Olena Tankyevych for specific research purposes.
